# “I don’t Teach Violence, I Teach Self-Control”; The Framing of Mixed Martial Arts Between Mental Health and Well-Being

**DOI:** 10.3389/fsoc.2021.750027

**Published:** 2022-01-17

**Authors:** Lorenzo Domaneschi, Oscar Ricci

**Affiliations:** University of Milano-Bicocca, Milan, Italy

**Keywords:** mixed martial arts, frame analysis, health cultures, body pedagogies, symbolic boundaries, qualitative analysis, new media, violence

## Abstract

This paper draws on conceptual and analytical tools from cultural sociology to analyze media representations of the MMA right after the murder of a twenty-year-old boy, that took place in a small village in central Italy by a gang of young men, two of whom frequented a MMA gym. While often characterized as violent and uncivilized, MMA has a core following of fans who watch and practice MMA out of an interest in the effects of the sport in terms of health and well-being. Through in depth qualitative analysis of  MMA media discourse offered by traditional and new media, this paper explores the way the MMA media constructs symbolic boundaries around different kinds of fights inside and outside the gym, through aesthetic and moral evaluations based on the hierarchical ‘distinctions’ between “violence” and “health” as possible outcomes of the MMA training process. Particularly, we carry out a discourse analysis based on Italian Newspapers, Magazines and Facebook groups dedicated to MMA, through which we frame the multiple representations of the discursive production built around the MMA in Italy. Our aim is to identify the different ways in which the discussion about this event provided narrative paths and points of view about the meaning of MMA, focusing on the reputational consequences concerning health, especially in its physical and mental expressions. This research may prove useful for scholars interested in MMA, culture, and sports media studies.

## Introduction

As martial arts are widely practiced, their effects on physiology, morphology, immunology and neurology should be further investigated in order to help people select the best discipline or style to achieve their goals (Bu et al., 2010). Indeed, Martial arts and combat sports (MACS) are believed to have a particularly significant educational potential, linked to several desirable values that provide positive models of health behaviours (Kotarska et al., 2019). For example, a notable contribution in terms of empirical research is the recent monograph by Fuller and Lloyd (2019). However, although these authors have explored the many benefits of physical training within different martial discipline systems, the sociological problem of health remains an extremely complex issue to conceptualize, especially from a cultural perspective (Channon et al., 2020).

Thus, the relationship that intertwines MACS cultures with issues of health and well-being is not only variously articulated but also, often, ambivalent (Blue, 2017; Kotarska et al., 2019). This, in fact, is mainly due to the issue of categorization and classification of individual disciplines and the related styles according to their effects on different body systems and levels of contact (Bu et al., 2010), as well as ways of standardizing assessment criteria in terms of health effects for individual martial arts. In short, what is at stake is precisely the construction of different competing philosophies and pedagogies of health within the huge spectrum of MACS (Jennings, 2014).

In this paper, therefore, we try to analyse the issues of health and well-being inside a particular martial sector of Mixed Martial Arts, starting from the discursive construction of its disciplinary boundaries. In fact, Mixed Martial Arts (MMA) is a controversial sport, since the health effects for practitioners–and spectators–have been the subject of controversy and debate since its inception (Andreasson and Johansson, 2019). Concerns, in fact, have mainly focused on the violent nature of MMA and, consequently, the related training pedagogies that formed its background. This point of view, therefore, allows the question of health as a controversial cultural form to be addressed directly. As we try to articulate in the following, unpacking the approach of MMA media from the perspective of health pedagogies allows us to see how some of their “cultural” work can lead to the social construction of a particular definition of health and well-being.

In the case of MMA, which is a recently developed sport, members of the media are in many ways responsible for this construction, invocation, explanation and representation of a pedagogical system. The MMA media classify, in fact, the “good” and the “bad”, the “beautiful” and the “strange”, the “insufficient” and the “excessive”, and thus stipulate the terms for their classification (Brett, 2017). This evaluative work of MMA media creates an entire symbolic world of discourse that operates outside of the fights themselves, a world that is eventually invaluable in understanding MMA as a pedagogical system.

To this aim, the paper is structured in three sections: the first part of the article frames the theoretical landscape in which we insert our proposal, declining the concept of cultivation (Pedrini and Jennings, 2021) from the point of view of the analysis of the discourse and social representations of the media that are responsible for presenting MMA in Italy to the mass audience and to the smaller circle of fans of the sector. Here we discuss how the (un)healthy pedagogies of martial arts and combat sports are framed in the specific case of MMA. The second part presents the boundaries of the research design, illustrating the specific case study we chose for our analysis and discussing the methodological choices in terms of data collection and analysis. The third part, in which we present the empirical results, is divided in two: an initial presentation and discussion of the framing operations of MMA by the legacy jurnalism; then, a second part of in-depth analysis of the discussions by experts and fans of the sector, occurred in UFC Italia, the most important Facebook group focused on MMA in Italy. In conclusion, we introduce a few questions implicated in our article about the relationship between health and martial pedagogies and address future possibilities for more research to be conducted in this line with a media-framing lens.

## Framing Unhealthy Pedagogies: The Case of MMA

In his seminal work on the genesis of a bodily pedagogy within a boxing gym, Wacquant (2004) focused in depth on the “expertly savage” practices that lead towards the transformation of body and soul into a fighter’s habitus. However, in the contemporary landscape of the “martial arts and combat sports” (MACS), beyond a myriad of systems of embodied movements, there is also a diffuse will to knowledge (Foucault, 1972) which transpires in an assorted Universe of philosophies and pedagogical systems that constitute, accordingly, diverse objects of disciplinary knowledge therefore identified as specific martial arts or combat sport.

In this sense, analyzing the “light” and “dark” sides of martial pedagogies (Jennings, 2019), it becomes significant to keep an eye also on the discursive procedures through which the content of these disciplines is publicly discussed and framed. It is crucial to understand such pedagogies, therefore, not only as systems of enacted practices but also and simultaneously as discursive formations (Foucault, 1972) that progressively establish bodily health and well-being as the goal of such practical, everyday processes of cultivation (Pedrini and Jennings, 2021).

Notably, within martial arts studies (Bowman, 2018) the question “how might martial arts and combat sports be good or bad for health?” has begun to be posed and discussed. However, on the one hand, the question has only been marginally addressed as a by-product of the broader discussion on the topic of violence and the processes of sportization of combat practices (Elias and Dunning, 1986; Bottenburg and Heilbronn, 2006; Spencer, 2009). On the other hand, more recently, the issue of practitioners’ health and well-being has been directly addressed (Jennings, 2014; Fuller and Lloyd, 2019) while still remaining within a framework that encompasses health as an embodied dimension.

However, while it is certainly important to overcome a reductionist definition of health, locked within the physiological and biomedical paradigm, in order to investigate the practical and embedded dimension of practices (Pedrini and Jennings, 2021), in the same way, we consider it equally crucial to investigate the construction of “(un)healthy pedagogies” (2021: 5) from the perspective of their discursive production, thus unfolding the very procedures of public definition of health (included what is excluded from such a definition), within the worlds of MACS.

In short, from this point of view, it is relevant to ask how the cultural legitimacy of a sporting practice is discursively constructed, starting from the conflicting representations of its constitutive rules (Coulter, 2009) regarding especially the methods and contents of its teaching.

This aspect is particularly crucial in the case of MMA in contrast with a combat sport such as boxing, for example. As Brett vividly stated, in fact: “while the legitimacy of the craft of boxing is almost taken for granted, for those outside this sphere of “internal legitimacy” of MMA, treating violence as an artistic and aesthetic endeavour may strike them as counterintuitive, offensive, or even morally wrong.” (2017: 16).

Similarly, while the pedagogical procedures and devices of boxing are by now institutionalised and framed in a consolidated media imaginary (Sheard, 1997; Rhodes, 2011; Vogan, 2020), in the case of MMA the social representations of their pedagogies are still polarised and debated. In fact, they usually split between a vision on the part of casual spectators more attracted to the taboo aspects of transgressing rules and conventions; and that of connoisseurs and heroes of the field, committed to presenting their work in the gym as an ongoing “testing the efficacy of fighting styles and techniques, legitimising some while debunking others.” (Brett, 2017: 17).

From this point of view, the case of media framing of pedagogies within MMA is particularly relevant precisely because their difference in cultural legitimacy with of a sport such as boxing can be interpreted in the light of their different framing in traditional and non-traditional media. In fact, most of the growth in popularity of MMA has occurred in the absence of primetime broadcast television opportunities (Frederick et al., 2012), and little coverage from ESPN (Martin et al., 2014). Consequently, much of what the mass public can learn about MMA necessarily comes from the limited, but often spectacularized coverage of the mainstream media; at the same time, however, MMA ‘connoisseurs’ often look to in-depth and very specialised online sources of information.

Finally, it is precisely in these differentiated venues and through these diverse forms of representation, that those “objects of disciplinary knowledge” of martial arts and combat sports are publicly reproduced and differentiated. Hence, these discursive formations become a relevant part of the process of cultivation (Pedrini and Jennings, 2021) of different audiences (Brett, 2017), inside and outside gyms: that is to say, a process of granting certain meanings of health and well-being as specific by-products of each martial discipline.

Therefore, when we depart from the biomedical and psychological paradigm to define objects such as health or well-being, we need to analyse the specific empirical procedures through which these objects of knowledge (Foucault, 1972) are produced within specific discursive arenas. In fact, by analysing in depth how the specific rules of the pedagogical game of each martial discipline are discussed, criticized or justified, we can reconstruct the boundaries through which the object of “health” is thought as an effect of truth (Foucault, 2017) within such particular discursive field. Finally, it will be this unique versions of health and well-being that will constitute the pedagogical goal of the discipline, consequently adapting the *cultivation* of the bodies of its disciples (Pedrini and Jennings, 2021).

Certainly, in the current landscape of sociological studies, there is a significant amount of literature devoted to exploring the topic of media framing in the context of combat sport (Messner & Solomon, 1993; Hutchins and Rowe, 2012), and there is also an emerging and increasingly consolidated sociological literature dedicated to MMA (García and Malcolm, 2010; Bottenburg and Heilbronn, 2006). However, there is a relevant lack of studies that explicitly focus on the intersection of these two issues, albeit with some relevant examples (Naraine and Dixon, 2014; Brett, 2017). However, while these works reveal the variety of investigations within the sphere of media framing and sport, they do not contribute much to our understanding of the effects of MMA media framing in fabricating (un)healthy pedagogies.

Therefore, the purpose of this research is to examine such framing in the context of MMA in the Italian context^1^, thereby extending the scope of media framing to this specific martial discipline within modern MACS.

### Research Design

On the night of September 6, 2020 Willy Monteiro, a 20-year-old cook, is killed with kicks and punches in Colleferro, a town of 20,000 people in the province of Rome. The investigations very quickly identify those responsible for the murder in four boys, two of those regularly attending an MMA gym in the same town. This event caused a great media clamor in Italy, which allowed the discourse on MMA to be at the center of the public debate for weeks. Accordingly, exploring the media framing of MMA attached to such a particular case allows us to call in question how the social construction of public knowledge of this martial art has relevant effects in the process of producing (un)healthy pedagogies.

The first part of our research focuses on analyzing how MMA’s presence was framed in the early days of the journalist’s coverage after the murder. The second part delves into how a community of MMA amateurs, gathered in the most important Facebook group dedicated to MMA in Italy, talk about the possible responsibility of this particular martial discipline in the commission of the crime. The first part of our media analysis focuses on what are generally referred to as legacy media: outlets of traditional journalism, generally paper-based, and also online platforms owned by the same traditional press. The data of this first round of analysis is taken from a range of news outlets accessed via the Internet. The sites were located through Google Italia searches of MMA and Colleferro from September 7, 2020 to September 11, 2020.

The websites returning hits from these searches included various different media outlets, such as national, regional, and local And the online services of local newspapers and television shows; sport and MMA-specific news websites. We held these various different news outlets to be significant sites through which public discourse about the Colleferro’s case takes form and begins to circulate.

All this first set of data thus gathered were sourced and translated into English. We then used a thematic analysis approach (Brawn and Clarke, 2019) to code the data. After identifying a broad range of themes pertinent to these theoretical issues, we eventually reduced these into three overarching analytical categories, which will be detailed in the next paragraph.

The second part of our media analysis focuses on analyzing the discussion of the Colleferro case on the most important Italian Facebook group dedicated to mixed martial arts in Italy: UFC Italia. The choice of mapping the discourses on this Facebook group allows us to understand how the themes proposed by the traditional press have been received, deepened, and eventually rejected by a group of MMA fans in the sense illustrated in the previous paragraph.

We selected this particular group as a significant site for a case study after 2 months of analysis of digital discourses related to the murder. Although these discourses have developed in all major digital platforms (Twitter, Instagram, Youtube, Reddit), we eventually selected Facebook as the most significant platform where to map this type of discursive production, especially through the participation in thematic and closed groups^2^. Particularly for potentially very sensitive topics, such as people’s physical and mental health, the closed and private group allows people to express themselves more easily, feeling more protected by a higher level of privacy (Bradshaw et al., 2020). UFC Italia, in particular, is a private Facebook group created on September 20, 2008; it is now composed of 21,997 members and it is actually the largest Italian group dedicated to this discipline on this platform. We selected all the posts dedicated to the event of Colleferro produced from the date of the murder until 4 months later. There were eight posts analyzed with a total of 2236 comments.

We analyzed comments based on media frame analysis (Scheufele, 1999), a research method that interprets the often implicit–meaning–of textual data through a process of systematic coding and identification of recurring analytic categories or frames. The frame, in this context, can be understood as a set of interpretive packages that give meaning to a problem, framing some aspects of reality and neglecting others (Gamson and Modigliani, 1989). We thus examined all the comments to the posts, to identify the different ways in which users make sense of the killing of Willy Montero, trying to highlight the discourses related to mental health and the potential effects of martial discipline on well-being.

### Framing the Colleferro Murder: Old Media Scenario

The murder at the center of our article takes place around three in the morning of September 6, 2020. The first newspapers to break the news are therefore the Sunday online editions, since the paper editions did not have the material time to publish the news until the following Monday, September 7. Since the very first articles published, it was clear that the attendance of an MMA gym by two of the four components of the beating is a relevant criterion of newsworthiness. In the first days after the event we can clearly see three trends in reporting this event.

The first one is when the attendance of the MMA  gym is put at the center of the news, but in this occasion the newspaper articles were not built on a strong condemnation of the discipline. Yet, the event serves almost as an excuse to explain some general features of this discipline, until then largely unknown to most of the Italian public. As we saw in the introduction, in fact, most of the growth in popularity of MMA has occurred in the absence of primetime broadcast television opportunities, therefore the mainstream press essentially took this opportunity to inform the general public of the very existence of this martial discipline.

An example of this first descriptive kind of framing is given by the following three different articles from the “Corriere della sera”, “La Repubblica”, and “Wired.it”^3^ published on September 7 and September 8:

Wrestling and Thai boxing: this is Mma, the sport of the brothers arrested for the murder The Mixed Martial Arts, practiced by the Bianchi brothers, accused together with Mario Pincarelli, 22, and Francesco Belleggia, 23, of having massacred with punches and kicks a young twenty-one year old who had intervened to defend a friend [these Martial Arts] come from Brazil and are a sport that mixes elements of many different disciplines. (La Repubblica September 7, 2020).

What are MMA, mixed martial arts?

The answers to all the questions about Mma, the mixed martial arts of which there is much talk after the killing of Willy Monteiro Duarte in Colleferro (Wired, September 8, 2020).

The Bianchi and Mma, what is the fighting art practiced by the two brothers arrested for the death of Willy Monteiro.

The brothers Marco and Gabriele Bianchi arrested in Colleferro are practitioners of the discipline in which punches, kicks, wrestling holds are allowed. “In the gym, there is no racism” (Il Corriere della Sera September 7, 2020).

This very neutral frame, however, is soon overridden by one that is decidedly more guilt-ridden. In the following articles, for example, violence already appears as almost the main by-product in teaching MMA:

MMA, what it is and why it’s so dangerous. “But on the street, violence is banned.”

For a few days now we have been talking about Mixed Martial Arts, MMA for short, the discipline of which the young brothers Marco and Gabriele Bianchi, arrested with two other boys for the murder of Willy Monteiro Duarte in Colleferro, were fans. But what is MMA, why is it so dangerous and what are its rules? (Leggo.it September 8, 2020)

Mma, what it is and why it is considered a violent and dangerous sport With the murder of Colleferro have become synonymous with violence and social degradation. What are the MMA, also known by the name of Mixed Martial Arts (Notizie.it September 8, 2020).

These articles operate by linking the idea of physical “danger” and “social degradation” to MMA world, assuming violence as one of the most relevant components of MMA pedagogy, even though they do not fully embrace the idea that MMA is a sport that is responsible for what happened in Colleferro. This sort of framing is also embedded by a series of articles that, taking almost for granted a part of MMA’s responsibility in the incident, work explicitly fabricating a polarization between the dangerous and physically worrying world of MMA, on the one side, and the “brave and generous” world of honest citizens, on the other side. Interestingly, though, most of these articles actually take their cues from a Tweet by the editor of “La Stampa”, Massimo Giannini.

Hello #Willy, brave and generous boy. But now, having punished the two exalted men who massacred him, do we want to ban certain “martial” disciplines and close the related gyms?

Soon after, this tweet also becomes a newspaper article, on the newspaper edited by the author of the tweet:

“Banning violent sports,” online controversy erupts (La Stampa September 8, 2020).

Following this article, essentially based on the tweet published by the director of the same newspaper, we see the publication of several articles which, while working on dismantling the previous thesis about MMA effect on good citizens physical health, eventually raised on mainstream media the debate around the relationship between MMA teaching and physical violence.

Colleferro, identifying martial arts with violence is unfair. In fact, the opposite is true (Il Fatto Quotidiano September 10, 2020).

Willy and the demonization of martial arts, the crazy debate that wants to ban MMA (Il Riformista September 9, 2020).

After punishing the two brutal men who massacred willy, do we want to ban certain “martial” disciplines and close their gyms?” Massimo Giannini’s tweet triggers online controversy–“if a carpenter commits a murder, do we criminalize all carpenters?–“there is no connection between mixed martial arts and those two morons”–in the gym of the two arrested: “this is not the fight club” (Dagospia August 8, 2020).

The fact that the “La Stampa” article obtained basically only criticism is particularly interesting, especially considering that, as we’ll see in the next paragraph, the same argument treated in “La Stampa” article will receive instead higher consideration in the Facebook group of MMA fans.

Another way in which the connection between MMA teaching procedures and the tragic event is constructed is through the interview of the privileged witnesses or experts. This role is offered in the first days of the journalistic coverage by two kinds of people: gym instructors and some of the most famous MMA Italian fighters.

Colleferro, the MMA champion Di Chirico: “Fighter? They look more like poachers. They have nothing to do with sport”. (Il messagero September 9, 2020).

Sakara: “Colleferro? Rotten apples. But the MMA are not a scapegoat".

“Legionarius” after the murder of young Duarte: “In 4–5 heavier against one, what mentality is that? Young people need teachers, examples, values. And in this sport do not look at McGregor but Miocic”. (La gazzetta dello sport September 8, 2020).

Death of Willy in Colleferro, the MMA champion Vettori: “4 cowards against 1”. (Le Iene September 8, 2020).

As we can see, all these interviews are roughly based on the same script: the fighters claim that “they” (a general they, here meaning the traditional press) are criminalizing MMA, but the responsibility lies with the individual protagonists of the affair and not with the martial art as a system of teaching. On the other side of such a picturing, though, mainstream media manage to tell the role of the teacher in this story, through interviewing the teacher of the two Bianchi brothers of, Luca Di Tullio, who is very willing to give interviews in the days immediately after the incident.

Colleferro, the trainer of the attackers: “My MMA does not teach violence. I am saddened, I will go to Willy’s funeral”.

“I am not the master of killers,” says Luca di Tullio, MMA instructor of Marco and Gabriele Bianchi, two of the attackers of Willy, the young 20-year-old killed Saturday night in a beating. “MMA does not teach violence” continues the instructor visibly shaken by the event. (Il Fatto Quotidiano September 8, 2020).

Homicide Colleferro, in the gym of Willy’s attackers. The MMA instructor: “I teach non-violence, I don’t train killers".

Luca Di Tullio is the instructor of the MMA Academy in Lariano, the gym where Marco and Gabriele Bianchi trained, accused of the beating of Willy Monteiro Duarte, the 21-year-old boy who lost his life in Colleferro. “They all attack me but I am not the master of the killers, I teach discipline,” he repeats between tears. (La Repubblica September 8, 2020).

Then, by building the debate against the idea that MMA is somewhat equal to violence, both MMA champions and instructors reinforce the idea that MMA pedagogy is just the opposite of physical violence and uncontrolled use of the body. Thus, as their instructor says: “I do not teach violence, I teach discipline” he actually intended the latter as the exact opposite of violence, that is self-control of one’s own body and being able to defence oneself from the menace of uncontrolled ferocity.

In the next paragraph, we will see how these issues are discussed in the Facebook group at the center of our analysis. Particularly, we will see how the frame of the responsibility of the teacher is just one of those that arouses the liveliest discussions, and how the group will not offer an univocal vision of this topic, dividing into absolutory and guilt-ridden positions.

### Framing the Colleferro Murder: The Debate on Facebook Group “UFC Italia”

The first frame on which we will focus is centered on the medicalization of gym attendance, particularly on the assessment of supposed psychological fitness at the time of the beginning of classes. This is a theme proposed by many comments, some of which, such as the following, call for the input of scientific experts to assess as scientifically as possible who is attending a gym^4^:

We need to bring in studies on the physiology of ethics, neurocognitive area, neurophysiology of aggression, behavioral neurobiology, etc. Personal defense? Have you ever seen a seminar in a gym with a lawyer, a judge, a police commissioner, or a psychologist? Friends, until we get out of the “volemose bene^5^” we will always be unprepared for all this that we are experiencing^6^

Firstly, it is quite interesting how the medicalization frame has been almost completely absent from mainstream media coverage. In this case, what we can see is how although the discussion in the Facebook group necessarily takes place among enthusiasts of the discipline, this does not exclude the possibility of creating even more profound boundaries than those fabricated by the traditional media system.

In fact, MMA amateurs demonstrate here that they are indeed very familiar with all the issues that martial discipline can bring to the health and well-being of its participants. Actually, they are indeed more expert in create differentiations in such a field.

One good example here, is the theme of psychological help for athletes. It started from commenting the episode of the murder and then quickly extends to cover other issues, one of the most important being domestic violence:

Sorry, we must be serious: think about how many MMA related athletes get arrested for domestic violence. The case of the Bianchi brothers is just the tip of the iceberg.

Ok the Bianchi brothers committed murder, so now the media will be all over  MMA for a few days. Of course, now the media will piss us off, but how many cases of domestic violence do we see every year that have MMA fighters as protagonists? Maybe more in the United States, but we cannot deny that there is a problem of psychological health and welfare of life within the environment of MMA.

Yes of course this case is crazy, and we can’t judge all of MMA in light of a murder. But it’s not the first time we have heard of violence related to MMA, to me the most disgusting ones are those related to domestic violence. In the gyms there must be psychologists.

Following the comments of the group participants, what distinctly emerges is the visualizing the MMA pedagogical environment as straightforwardly unhealthy, especially psychologically. Although the discussion starts from the case of Colleferro murder, in fact, we can see how other details apparently unrelated to such an event come to light that help to delineate a generally not very encouraging picture. The mainstream mediatization of the murder of the Bianchi brothers may let think that it has been a somewhat isolated case in the landscape of MMA commentators and amateurs. Yet, dozens of comments focused on cases of domestic violence and abuse, that promptly appeared in the social media arena, starting from the discussion of the murder, portray a situation where psychological problems are widespread even when the mainstream media turn off the spotlight. The case of the Bianchi brothers thus acts here almost as a proper excuse to broaden the discourse to other aspects where MMA seems to generate health issues. Moreover, it helps to raise the question of the need for a presence of constant psychological support for athletes.

We see in the next comment, for example, how the request to have a psychological interview at the time of entry into the gym would thus favor the teacher’s work, so as to help him decide who can and who cannot attend. In this way, the teacher’s responsibility would be confirmed, but in some way also limited by the help of a psychological evaluation:

It would take (for any sport) a talk with a psychologist before starting to practice, this could also help coaches to know better the kids and avoid these episodes.

The second frame, link to the previous one, is the one focused on who or what to blame for the incident. This frame is divided into two subframes: one, narrower, that places the responsibility primarily on the teacher in the gym, and the second, more substantial, that discusses whether responsibility can be placed on the sport itself. The discourse on responsibility, whether it is directed on the teacher or on the martial art itself, is one of the places where we best observe the process of cultivation in action. It is here, in fact, in attributing whose responsibility it is that the practice of this discipline is framed as healthy and not harmful–that is, the opposite of physically dangerous and cruel. Among the few comments that tend to attribute at least a portion of responsibility to the teacher there are, of course, comments from teachers themselves, who passionately explain how in their daily practice they tend to try to limit gym attendance to certain individuals they considered dangerous:

I am an instructor of  MMA and I know all my boys, both good and not so good. The master as well as teaching the discipline must teach respect for people and respect between students. My teacher as well as being a great instructor is a mentor and a teacher of life. He has taken boys off the streets. He has made real men of value and respect with a few words and a lot of sacrifice and teaching.

As this kind of comments make clear, the idea of being “a teacher for life” stands exactly in front of the professional ability to transform “bad boys”—that is individuals dangerous for others and for themselves–in “real men of value” teaching them “respect for people”. Again, but even more detailed, the achievement in participating in MMA training is precisely to reach a “healthy” place defined as the opposite to the mainstream discourse of uncontrolled cruelty and unsafe state of mind which is the prevailing definition of bad western citizens.

In contrast, comments focusing on the responsibility of the sport itself are numerous and have a variety of nuances. Nearly all of them respond to other comments that propose an essentially absolutory position, where the sport is treated neutrally, as in the comment below:

What in the world does martial arts have to do with anything? What? These guys are gutless cowardly bastards! No matter what discipline they practice! I myself practice combat sports and am a respectful and decent person. As are a myriad of people who practice martial disciplines! Martial arts are about respect, honor, sacrifice, dignity, discipline.

These scum are not even worthy of the name they bear! They are a disgrace to MMA and all combat sports. I hope justice is served and they are disbarred for life from all sports.

In contrast to this opinion, we find dozens of comments that, sometimes very subtly, other times decidedly more directly, question the fact that the practice of this martial arts is not so neutral, and that perhaps it may have had a bearing on the training of the two murderers:

However, if one has attended combat sports gyms, he knows that, deep down, the criticisms that are coming are not so far from the truth.

You have to be realistic. Personally, in 15 years of experience with the gyms I have known many good guys but so many hotheads, starting with the coaches. in the gyms of MMA, boxing, k1 etc. if you make a survey of how many people had problems for complaints for attacks etc. you realize that from the outside they see us as we are. while we inside, do not want to see the reality as it is.

This is starting from the coaches, to the amateur boxers until you get to the professionals!!!

The professionals who have never had any problems with the law related to violence, you can count them on two hands. so this is it!

One interesting distinctions to solve this quarrel between the responsibility of MMA in creating unhealthy pedagogies is the one about the boundaries between “combat sports” and “martial arts”. One of the characteristics attributed to martial arts, as we have tried to show so far, is to teach, besides the art of combat, a philosophy of life, which instils respect for the opponent and to behave with dignity inside but especially outside the ring. Accordingly, martial arts would thus be a place where body and mind both benefit from a continuous training that tends to improve the behavior of those who adhere to the precepts that are taught. This assumption, which we have seen proposed–albeit in somewhat crude terms–in the first comment that we reported, is however challenged by those who argue that in reality MMA actually have a complex relationship with martial arts. The discussion here funnels into a series of nuances.

However, for those who stress this distinction, as recently suggested by Martínková and Parry (2021), MMA cannot be actually defined as a fully-fledged martial art, but is simply a combat sport. The absence of the component attributed to martial art would remove the spirit of respect for the opponent leaving just room for the veneration of violence:

There are Martial Arts and Martial Arts, don’t tell me that MMA doesn’t exalt violence and the overpowering of the opponent, Martial Arts are born for self-defense not to learn how to fight.

Let’s not confuse martial arts and combat sports. In the first one a sensei can also kick you out of the gym because he knows you use the art outside the gym. In the second let’s say he cares less about what you do outside the gym.

Combat sports in general certainly do not promote ethics, fellowship between human beings, philanthropy and compassion, but they do expose the animalistic part of the human being.

Here, once again, we find at work that work on boundaries through which a certain discursive partition is constructed between what is healthy defined on the basis of its categorical opposite. Unlike the case of traditional media, however, in which this partition occurred based on a more vague discourse external to the specific vocabulary of the field of MMA, in this second part something different happens. In fact, as we have seen, the specific skills of the practitioners, the multiple nuances they have to think about and describe their discipline are put at the service of a boundary work that is even more sophisticated, but that in the final analysis only traces this type of partition. The gain in terms of health and well-being activated by martial pedagogies, in short, is such only if it adheres to a certain regime of truth according to which it is healthy that which moves away from the dimension of physical danger and lack of mental self-control. The extreme cases of domestic violence, of the psychological control needed within gyms for more dangerous subjects or, finally, of the exclusion of MMA from the authentic spirit of “true” martial arts only confirm this kind of discursive construction of healthy pedagogies within the field of MMA.

## Conclusion

This article seeks to show the analytical value of cultural sociology for MMA–and martial arts and combat sports more generally–as well as the empirical value of MMA for cultural understanding of issue such as health and well-being. By analysing the critical, aesthetic and cognitive potential of MMA media framing (Naraine and Dixon, 2014; Brett, 2017), this article highlights their pedagogical and evaluative role, as well as the moral apparatus created both inside and outside of such a controversial sport (García and Malcolm, 2010; Andreasson and Johansson, 2019). Indeed, as we have tried to show, implicit and powerful boundaries, hierarchies and moral and pedagogical values are erected around particular types of martial disciplines, thus transforming issues of health and well-being into specific cultural stakes (Jennings, 2019; Channon et al., 2020). Thus, not only does the cultural sociology approach provide an understanding of the health pedagogies inscribed in MMA, but at the same time MMA, as a controversial and specific martial discipline, can deepen our understanding of health and wellness cultures.

Particularly, we have seen how the dramatic case of Colleferro has constituted the occasion for a public debate on MMA in Italy. Obviously given that this discussion developed as a result of a murder there were reasonable expectations that the general connotation of the discourses were predominantly pessimistic. Despite this, we were able to observe an interesting difference between the coverage of the press and how this theme has been handled in the discussion between fans. While the mainstream coverage focused on producing a general blaming of the martial art as a multiplier of violent and unhealthy pedagogies, the community of fans delved more deeply into various aspects of MMA’s relationship to the physical and mental health of its participants. Notwithstanding the fact that one might expect a generally absolutist tone towards MMA from those who are its fans–though partially this has certainly been the case–this event has been an opportunity to question and explore various issues of this combat sport from those who are supposed to be more inclined to defend it (Frederick et al., 2012; Zembura and Żyśko, 2015). It was in fact the potential well-being that MMA would fail to bring to their followers that eventually leads many commentators to question the belonging of MMA to the family of martial arts, which in contrast are mostly perceived as helpful places in which to experience physical and mental growth (Fuller and Lloyd, 2019).

Although of course we have to assume that these comments were influenced by the specific news event from which the discussion arose, such judgments coming from fans, experts and connoisseurs of the discipline express neatly how the very definition of health and wellbeing is perceived by practitioners as an internal stake linked to the boundaries of the martial discipline itself. Accordingly, we discussed how the (un)healthy pedagogies of martial arts and combat sports are framed in the specific case of MMA. Analysing the discursive construction of such pedagogies, in fact, allow us to illuminate how creating bodily health and well-being is the goal of such practical, everyday processes of cultivation (Pedrini and Jennings, 2021). Even though this process is composed by moments of enactment of practices, we have tried to show how it is also inevitably caused by the discursive procedures of public definition of health, within and without the worlds of MACS.

Thus, it will be this distinctive definition of health and well-being that will establish the pedagogical goal of the discipline, consequently adapting the practices of disciplining the bodies of its adherents (Pedrini and Jennings, 2021). In this line, the discourse on responsibility of obtaining (or not) such a goal, whether it is set on the teacher or on the martial art itself, is one of the places where we best observe such process of cultivation in action.

Hence, understanding how health is framed in relation to a specific disciplinary system enlarge and enrich cultural approach to health based on embodiment and practice (Blue 2017; Pedrini and Jennings, 2021), while calling in question the approach that is usually grounded on the idea of “social determinants” of health (Baum 2008; Cynarski et al., 2017). The approach we have tried to address, in fact, has been to investigate the socio-cultural-martial context in which health behaviors are evaluated and produced, exploring the availability of various health resources including discursive fields promoting–or obstructing–articular healthy or unhealthy pedagogies with their attached ideal notions of what a healthy citizen might be (Thompson and Kumar, 2011).

## Data Availability

The original contributions presented in the study are included in the article/Supplementary Material, further inquiries can be directed to the corresponding author.

## Footnotes

1 At the beginning of 2010, FILA (International Olympic Wrestling Federation recognised by the IOC–International Olympic Committee) introduced amateur MMA, which came under the management of IMMAF (International Mixed Martial Arts Federation) in 2014, which is supported and funded by the UFC. In Italy, MMA is managed by the Italian Grappling Mixed Martial Arts Federation (FIGMMA) which is internationally recognised by IMMAF and in Italy by the CONI Federations FIJLKAM (Federazione Italiana Judo Lotta Karate Arti Marziali) and FIWUK (Federazione Italiana Wushu Kung Fu).

2 For an overview of the issues concerning doing research on closed groups and ephemeral content see Boccia Artieri, Brilli, Zurovac (2021)

3 “La Repubblica” and “Corriere della sera” are the two main national newspapers in Italy. Wired.it is the Italian version of Wired.com.

4 All comments reported here are taken from posts dedicated to the murder of  Willy Monteiro published in the Facebook group UFC Italy. The link of the closed group is https://www.facebook.com/groups/25610244838

5 “Volemose bene” is an expression of the Roman dialect that indicates the attitude to pass over problems, not to create others.

6 One must consider that this is a group dedicated to MMA and UFC fans, so the prevailing opinion which is an absolutist one for the martial art should not come as a surprise. However, as we shall see, many critical voices have been raised.
